# Supplementary material for: Revealing the Angiogenic Signature of FH-Deficient Breast Cancer: Genomic Profiling and Clinical Implications
Source: Cancers (Basel). 2025 Sep 9;17(18):2942. doi: 10.3390/cancers17182942 (PMC12468410; doi:10.3390/cancers17182942)
Supplement: Supplementary file 1 [file cancers-17-02942-s001.zip › Supplementary Figure S1.pdf]

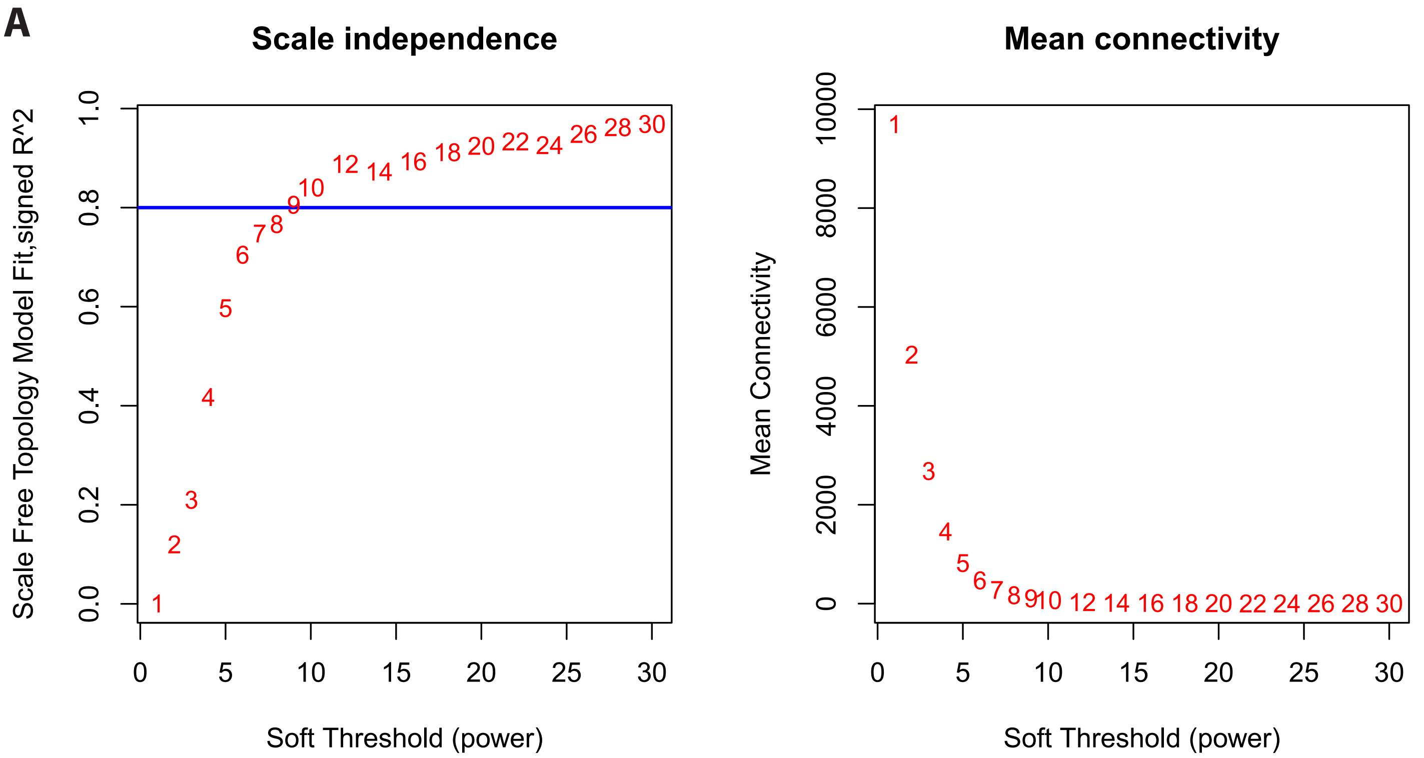

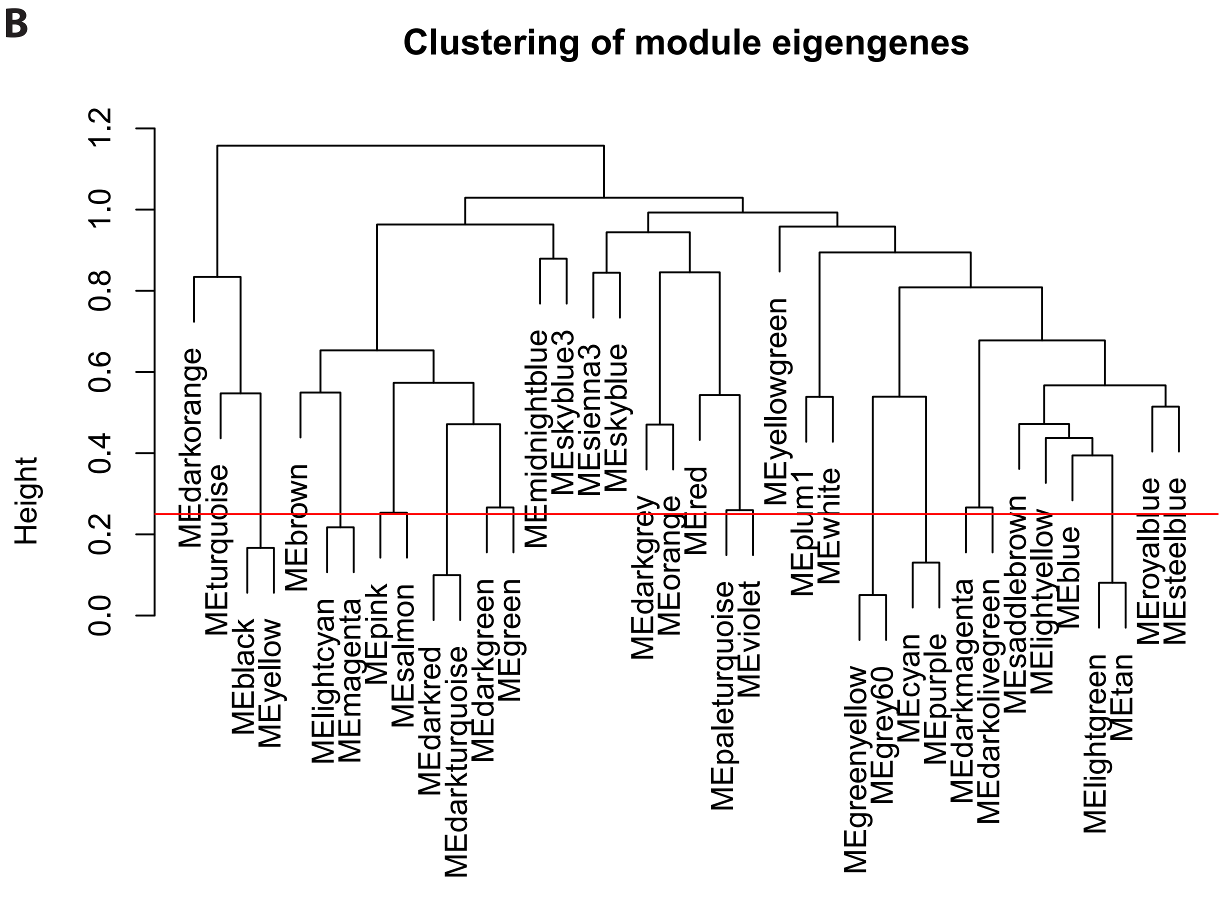

Supplementary Figure S1: **Selection of soft-thresholding power and module merging in WGCNA**. (**A**) Analysis of the scale-free topology fit index (left) and mean connectivity (right) across a range of soft-thresholding powers. A power of 12 was chosen, as the recommended power for signed network which the scale-free topology fit index exceeds 0.8 (blue line). (**B**) Clustering dendrogram of module eigengenes. Modules were merged at a cut height of 0.25, as indicated by the red line.
